# Antitumor Effect of Ruthenium(II) with 2‑Mercaptothiazoline Ligand Complex in Murine Melanoma Model

**DOI:** 10.1021/acsomega.6c01894

**Published:** 2026-08-04

**Authors:** Gabriela Fernandes, Arthur Barcelos Ribeiro, Matheus Reis Santos de Melo, Iara Silva Squarisi, L. H. D da Silva, Monize Martins da Silva, Sonia de Assis, Alzir Azevedo Batista, D. C. Tavares

**Affiliations:** † 92917 Universidade de Franca, Avenida Dr. Armando Salles Oliveira, 201 − Parque Universitário, Franca, São Paulo 14404-600, Brazil; ‡ 681196 Universidade Federal de São Carlos, Departamento de Química, Rodovia Washington Luis s/n Km 235, São Carlos, São Paulo 13565-905, Brazil; § Georgetown University, Department of Oncology, Lombardi Comprehensive Cancer Center, Washington, District of Columbia 20057, United States

## Abstract

Ruthenium-based complexes have been explored as promising alternatives in antineoplastic therapy due to their enhanced selectivity for cancer cells. In this context, the present study evaluated the antitumor potential of the ruthenium complex [Ru­(mtz)­(dppe)_2_]­PF_6_, named RuMTZ, in C57BL/6 mice bearing B16-melanoma, as well as its toxicity. In addition to tumor weight and mitotic frequency, cleaved caspase-3, γH2AX, and MAPK/ERK expression levels were analyzed by immunohistochemistry. The toxicity of the treatments was assessed through alterations in body and organ weight, biochemical parameters of liver and kidney function, and genotoxicity in bone marrow cells. RuMTZ led to a significant reduction in mitotic frequency and tumor weight, with a tumor growth inhibition rate equivalent to 94.9%. The antimelanoma activity of RuMTZ was accompanied by increased levels of cleaved caspase-3, γH2AX, and MAPK/ERK. These data indicate that RuMTZ induced DNA strand breaks, which may be, at least in part, related to the induction of caspase- and ERK1/2-mediated apoptosis. RuMTZ treatment, unlike cisplatin, showed no weight loss or impairment of liver and kidney functions. However, similar to other metallopharmaceuticals, it caused myelosuppression, evidenced by the absence of erythrocytes. These findings support the promising antitumor activity of RuMTZ, indicating its potential as a metallodrug.

## Graphical abstract

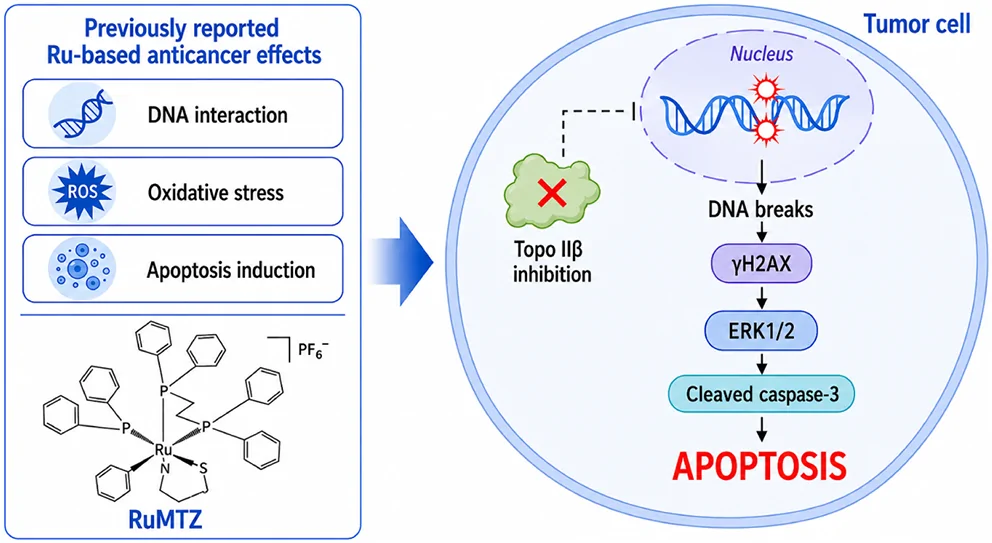

## 1 Introduction

Empirical evidence supporting the therapeutic potential of metal-based compounds in cancer treatment has been known for centuries, however, their relevance in cancer therapy only gained prominence following the demonstration of cisplatin’s antitumor activity in 1969.^1,2^ Despite its clinical success, cisplatin and other platinum-based agents exhibit limited tumor selectivity and dose-limiting toxicities, including nephrotoxicity, immunosuppression, and hypersensitivity reactions. These limitations, together with the emergence of resistance, have motivated the search for alternative metal-based therapeutics.^3,4^

Ruthenium complexes have emerged as particularly attractive candidates because their redox properties, ligand-exchange kinetics, and coordination chemistry can be rationally tuned to modulate cellular uptake, biomolecular interactions, and toxicity profiles. Several ruthenium compounds have advanced to clinical evaluation, underscoring their translational potential, especially in settings where platinum resistance compromises outcomes,^5–7^ In addition, Ru­(III) complexes have often been proposed as prodrug-like species that may undergo activation to Ru­(II) in tumor-relevant microenvironments, enabling multitarget interactions and diverse anticancer mechanism.^8^

The incorporation of ligands into metal complexes can further enhance antitumor activity. Mercapto molecules have been used as ligands to produce compounds with various biological activities, primarily due to their inherent anticancer properties and the potential for synergistic effects when coordinated with metals,^9–11^ In addition, Ru–phosphine-based compounds have been investigated for their cytotoxicity, since the phosphine group increases the lipophilicity of the complex, facilitating its entry into cancer cells. Moreover, the literature reports that these compounds can cause mitochondrial damage, triggering apoptosis.^12^

Although the mechanisms of action of ruthenium­(II) complexes are not fully elucidated, accumulating evidence supports a multitarget profile involving interactions with DNA and DNA-processing pathways, as well as modulation of stress- and survival-related signaling networks. Such multitarget behavior may be advantageous in heterogeneous and therapy-resistant tumors but also highlights the importance of integrated preclinical studies that combine efficacy with mechanistic and safety end points.^10,11^

Within this framework, Silva and colleagues^10^ previously demonstrated the selective *in vitro* cytotoxic activity of a ruthenium­(II) with a 2-mercaptothiazoline ligand complex ([Ru­(mtz)­(dppe)_2_]­PF_6_; Figure 1), named RuMTZ, against several human tumor cell lines, including lung, triple-negative breast adenocarcinoma, estrogen-dependent epithelial cancer, prostate carcinoma, and hepatocellular carcinoma. Subsequently, our group demonstrated selective cytotoxicity of RuMTZ in human (A-375) and murine (B16–F10) melanoma cells, accompanied by morphological alterations, increased sub-G0/G1 population, and impaired migration. Mechanistically, RuMTZ increased γH2AX (a marker of DNA damage response) and cleaved caspase-3 (a key executioner of apoptosis), suggesting that DNA damage signaling and apoptotic pathways contribute to its anticancer effects in melanoma models.^13^

**Figure 1:**
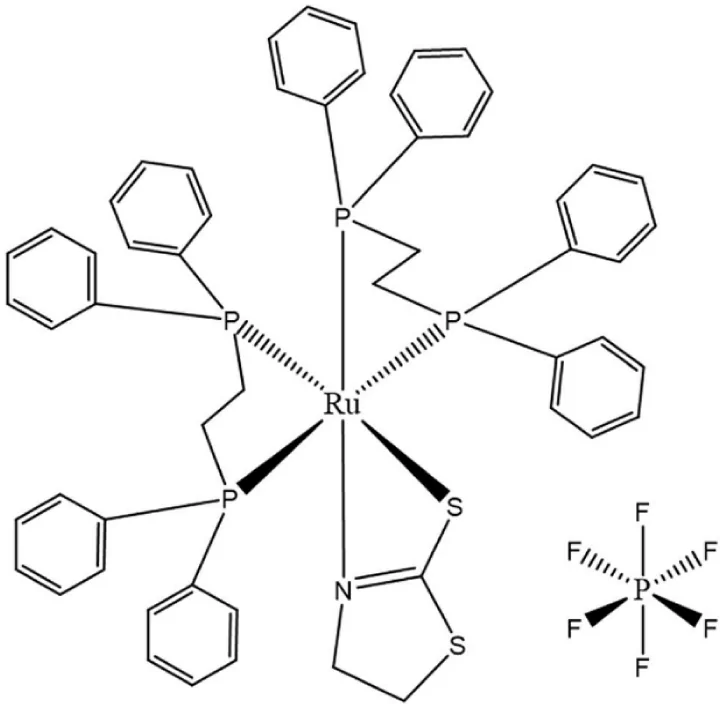
Chemical structure of [Ru­(mtz)­(dppe)_2_]­PF_6_.

Melanoma remains a clinically relevant target due to its aggressive behavior and metastatic potential. In the United States, it is estimated that in 2025 approximately 104,960 new cases will be diagnosed and 8,430 deaths will occur.^14,15^ At the molecular level, melanoma frequently harbors recurrent genetic alterations that converge on hyperactivation of the RAS–RAF–MEK–ERK (MAPK/ERK) cascade, including mutations in BRAF, NRAS, and NF1, as well as alterations affecting upstream receptor signaling.^16^ This pathway is strongly implicated in melanoma cell proliferation, survival, and progression, supporting the biological relevance of evaluating MAPK/ERK components in preclinical studies.^17^ Global climate change, combined with increased ultraviolet radiation exposure, has contributed to rising incidence and mortality rates of melanoma, thus posing a significant threat to public health.^18^

While in vitro findings provide important initial evidence of anticancer activity and support mechanistic hypotheses, positive in vitro results do not guarantee therapeutic efficacy in vivo, where pharmacokinetics, biodistribution, and tumor–host interactions can critically shape outcomes. Therefore, appropriate murine models are essential in oncology drug development to support efficacy evaluation and systemic safety assessment in a biologically integrated context.^19,20^ In this context, the aim of this study was to evaluate the antitumor potential of RuMTZ in mice bearing melanoma, along with its toxicity profile. In addition to assessing tumor weight and mitotic index, molecular markers associated with the DNA damage response (γH2AX), apoptosis (cleaved caspase-3), and the MAPK/ERK signaling pathway were analyzed to better understand the mechanisms of action involved. Toxicity was monitored through alterations in body weight, liver and kidney function, and genotoxicity.

## 2 Materials and Methods

### 2.1 Obtaining the Ruthenium Complex – RuMTZ

RuMTZ ([Ru­(mtz)­(dppe)_2_]­PF_6_ – 2-mercaptothiazoline-di-1,2-bis­(diphenylphosphino)­ethane ruthenium­(II) hexafluorophosphate) was synthesized and characterized as previously described by Silva and colleagues.^10^ Briefly, the complex was obtained by reacting cis-[RuCl_2_(dppe)_2_] with the corresponding mercapto ligand in a 1:1 methanol/dichloromethane mixture under an argon atmosphere at reflux for 12 h, yielding a yellow solid in 82%. Structural characterization was confirmed by single-crystal X-ray diffraction (CCDC 2079011), IR spectroscopy, NMR ^1^H, ^13^C, ^31^P­{^1^ H}), and cyclic voltammetry (Epa = 1116 mV).

This ruthenium complex was dissolved in dimethyl sulfoxide (DMSO, Sigma-Aldrich, St. Louis, MO, USA; CAS 67-68-5; 1%) for use in the experiments.

### 2.2 Cell Line and Culture Conditions

B16–F10 murine melanoma cells, kindly provided by C. Costa-Neto (University of São Paulo, Ribeirão Preto, SP, Brazil), were used in this study. Cells were cultured in RPMI medium (Sigma-Aldrich) supplemented with 10% fetal bovine serum (Cultilab, Campinas, SP, Brazil), 1.2 g/mL sodium bicarbonate, 0.1 g/mL streptomycin, and 0.06 g/mL penicillin (all from Sigma-Aldrich), and maintained at 37 °C in a humidified 5% CO_2_/air atmosphere. Cell viability and counting were assessed using a Muse Cell Analyzer (MCH100106, Merck, Darmstadt, Germany), ensuring ≥95% viability prior to rodent implantation.

### 2.3 Animals

Male C57BL/6 mice, weighing approximately 25 g (body weight, b.w.), were obtained from the Animal Facility of the University of São Paulo (Ribeirão Preto, SP, Brazil) and allocated into experimental groups (n = 5 per group). For histochemical analyses, three animals per group were selected, with groups matched according to initial tumor volume to ensure consistency. Animals were housed in plastic cages under controlled environmental conditions (23 ± 2 °C, 50 ± 10% humidity) on a 12 h light/dark cycle, with food and water provided *ad libitum*. All animal procedures were conducted in accordance with institutional ethical guidelines for animal care and were approved by the Ethics Committee on Animal Use of the University of Franca (protocol no. 8761060220).

### 2.4 Experimental Design

Tumor induction and experimental design followed the procedures described by Carnizello and colleagues.^21^ When tumor volume reached approximately 100 mm^3^, which generally occurred 12 days after cell implantation, the animals received subcutaneous treatment once daily for five consecutive days, administered at a site 1.0 cm from the tumor.

The following control and treatment groups were established: negative control (NC, no tumor implantation); DMSO (with tumor implantation and treated with 1% DMSO vehicle); RuMTZ (with tumor implantation and treated with RuMTZ – 5 mg/kg); 2- mercaptothiazoline (MTZ, with tumor implantation and treated with MTZ ligand – 0.59 mg/kg); Ru­[Cl_2_]­dppe_2_)­PF_6_ (RuCl_2_, with tumor implantation and treated with RuCl_2_ – 4.78 mg/kg – without the MTZ ligand) and cisplatin (CDDP, with tumor implantation and treated with CDDP – 5 mg/kg). The RuMTZ and CDDP doses were selected based on the literature.^22^ For MTZ and RuCl_2_, the doses used in the treatments corresponded to the amounts of these ligands presented in 5 mg/kg b.w. of RuMTZ.

The tumor volume of the animals was measured daily throughout the experiment. Twenty-4 h after the last treatment, the animals were euthanized with thiopental sodium (0.84 g/kg; Cristália, Itapira, SP, Brazil). Tumors were excised from each animal, weighed, and stored for subsequent histopathological analysis. Immediately prior to euthanasia, blood samples were collected via cardiac puncture, and plasma was stored for subsequent biochemical assessment of nephrotoxicity and hepatotoxicity. The heart, liver, spleen, kidneys, and lungs were collected and weighed. Bone marrow samples were collected to assess the genotoxic potential of the treatments.

### 2.5 Assessment of Antitumor Activity

Tumor volume (mm^3^) was calculated using the following formula:^23^

$$V\left({\mathrm{mm}}^{3}\right)=\frac{{\left[\mathrm{length}\;(\mathrm{mm})\times \mathrm{width}\;(\mathrm{mm})\right]}^{2}}{2}$$

The tumor growth inhibition rate (TGIR) was evaluated according to Qin et al. (2009):^24^

$$\mathrm{TGIR}=\left(\frac{\left[I-T\right]}{I}\right)\times 100$$

where *I* corresponds to the tumor mass of the implant group (g) and *T* corresponds to the tumor mass of the treatment group (g).

Mitotic figure frequency, a marker of cell proliferation, was assessed in tumor samples. Therefore, excised tumors were fixed in 10% neutral formalin and embedded in paraffin blocks. From each tumor, five randomly selected 5 μm-thick sections were obtained, deparaffinized, and stained with hematoxylin and eosin. Mitotic figures were counted in five randomly selected fields per section at 400× magnification, resulting in a total of 15 fields analyzed per group.

### 2.6 Immunohistochemistry

Immunohistochemical analyses were conducted to better understand the signaling pathways involved in the activity of RuMTZ on melanoma. For this purpose, antibodies against the following proteins were used: cleaved caspase-3 [asp 175] (apoptosis); H2A histone family member X, γH2AX [ser 139] (DNA damage); and mitogen-activated protein kinase isoforms p42 and p44, p44/42 MAPK [ERK 1/2] (cell proliferation, differentiation and survival) (Cell Signaling, Danvers, MA, USA). To assess cleaved caspase-3 levels, tumor tissue sections (5 μm) were deparaffinized, rehydrated, and subjected to antigen retrieval. After blocking nonspecific binding and endogenous peroxidase, sections were incubated overnight at room temperature with primary antibodies. Detection involved incubation with postprimary and HRP-conjugated secondary antibodies, followed by visualization using DAB.^25^ For the assessment of γH2AX and MAPK/ERK expression, the procedures were conducted by the Histopathology & Tissue Shared Resource of the Lombardi Comprehensive Cancer Center, Georgetown University (Washington, DC, USA).

The tumor tissue sections were systematically prepared on the same batch of slides, counterstained with hematoxylin and photographed at 400× magnification under a bright-field microscope (Nikon Eclipse E200; Moticam 580INT camera, Nikon, Shinagawa, Tokyo, Japan).

The images were analyzed using ImageJ software with the IHC Toolbox training plug-in, as described by Shu et al.^26^ Results are expressed as optical density (OD), calculated according to Mustafa et al.^27^ using the following formula:

OD=log(maximumintensity/averageintensity)

### 2.7 Toxicity Assessment

Renal and hepatic functions were evaluated by measuring serum creatinine and alanine aminotransferase (ALT) levels, respectively, in addition to monitoring body weight changes and organ weights throughout the experiment. Analyses were performed using Mindray automated analyzers with enzymatic colorimetric assay kits.

The genotoxic potential of the treatments was assessed using the bone marrow micronucleus assay, conducted in accordance with the protocol established by MacGregor et al.^28^ and the guidelines outlined in OECD 474.^29^ Micronucleated polychromatic erythrocyte (MNPCE) frequencies were determined by analyzing 4,000 polychromatic erythrocytes (PCE) per animal, resulting in a total of 20,000 PCE per treatment group. To evaluate treatment-related cytotoxicity, the ratio of PCE to total erythrocytes (TE = PCE + NCE, normochromatic erythrocytes) was calculated based on the analysis of 500 erythrocytes per animal.

### 2.8 Statistical Analysis

The data obtained were statistically analyzed by analysis of variance (ANOVA) and Student’s *t* test for completely randomized experiments, with evaluation of the F statistic and the corresponding P values. When *p* < 0.05, Tukey’s test was applied to determine the minimum statistical difference for = 0.05. All statistical analyses were performed using GraphPad Prism software (version 6.0).

## 3 Results and Discussion

### 3.1 Assessment of Antitumor Activity

The results of tumor weight measurements after treatment with RuMTZ and control groups are illustrated in Figure 2. Animals treated with RuMTZ showed a significant reduction in tumor weight compared with those treated with the DMSO vehicle, with a TGIR of 94.90%.

**Figure 2:**
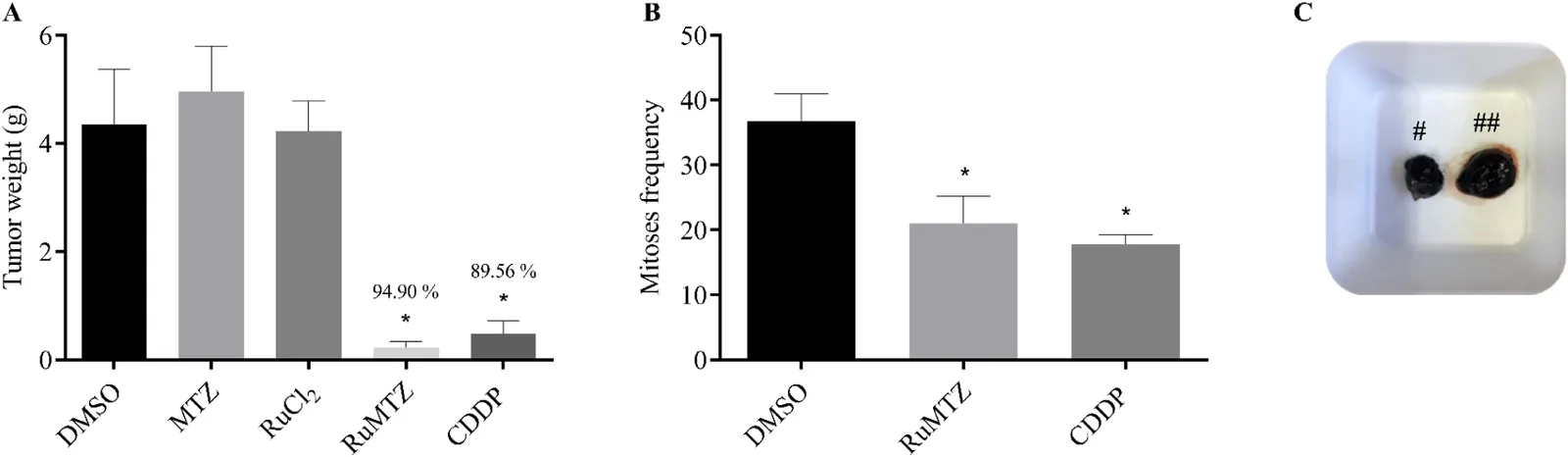
Assessment of antimelanoma activity in tumor-bearing C57BL/6 mice subjected to treatment with RuMTZ for five consecutive days and the respective control groups. (A) Tumor weight. (B) Frequency of mitotic figures (five random fields = total of 25 areas per group). (C) Tumor from an animal treated with RuMTZ (#) and an animal treated with the DMSO vehicle (##). DMSO – dimethyl sulfoxide (1%); MTZ – 2-mercaptothiazoline (0.59 mg/kg); RuCl_2_ – Ru­[Cl_2_]­dppe_2_)­PF_6_-cis-dichloro-di-1,2-bis­(diphenylphosphine)­ruthenium­(II) (4.78 mg/kg); RuMTZ – [Ru­(mtz)­(dppe)_2_]­PF_6_-2-mercaptothiazoline-di-1,2-bis­(diphenylphosphine) ethanoruthenium­(II) hexafluorophosphate (5 mg/kg); CDDP – cisplatin (5 mg/kg). Values are expressed as mean ± standard deviation (n = 5). *Significantly different from the DMSO group (*p* < 0.05).

For comparative purposes, CDDP, a reference platinum-based chemotherapeutic and historical benchmark for metal-based anticancer agents, was included to contextualize RuMTZ efficacy under the same experimental conditions. CDDP achieved an 89.56% inhibition of tumor growth (Figure 2A). In contrast, animals treated with the free ligand (MTZ) or RuCl_2_ did not differ from the vehicle group, indicating that the antimelanoma activity is associated with the intact RuMTZ complex rather than its isolated components. A representative comparison of tumor volume between a RuMTZ-treated mouse and a vehicle-treated mouse is presented in Figure 2C.

The frequency of mitotic figures in tumor tissues from the different treatment groups are illustrated in Figure 2B. Treatment with RuMTZ led to a significant reduction in mitotic frequency compared with the DMSO group, indicating decreased cell proliferation. Similar results were observed for the group treated with the metallopharmaceutical CDDP.

Therefore, RuMTZ exhibited antitumor activity in a murine melanoma model, as evidenced by a significant reduction in the weight, growth rate, and cell proliferation. These findings are consistent with the cytotoxicity data in melanoma cells previously demonstrated by our research group.^13^

Previous studies conducted by Silva et al.^6^ demonstrated that the ruthenium complex (Ru­(dmp)­(dppe)_2_]­PF_6_) also exhibited antimelanoma activity under experimental conditions similar to those of the present study. The TGIR obtained after treatment with (Ru­(dmp)­(dppe)_2_]­PF_6_) was approximately 79%, whereas RuMTZ presented an inhibition rate of 94.9%. In this context, the RuMTZ complex proved to be more effective. The observed difference is likely related to the specific mercapto ligand present in each complex. While [Ru­(dmp)­(dppe)_2_]­PF_6_, named RuDMP, contains a 4,6-diamino-2-mercaptopyrimidine (DMP) ligand, RuMTZ features a 2-mercaptothiazoline (MTZ) ligand.

Ru­(II) complexes bearing the MTZ ligand have been reported to display higher cytotoxicity than DMP-containing analogs in tumor cells.^10,30^ Structurally, RuDMP contains a 4,6-diamino-2-mercaptopyrimidine (DMP) ligand, whereas RuMTZ incorporates 2-mercaptothiazoline (MTZ). Differences in charge distribution and lipophilicity may influence cellular uptake and intracellular distribution; notably, RuDMP has been reported to be less lipophilic than RuMTZ, which could contribute to reduced membrane permeation and lower biological activity. Accordingly, variations in ligand-dependent properties such as lipophilicity and electronic effects are plausible contributors to the enhanced antimelanoma efficacy observed for RuMTZ.

To explore pathways associated with RuMTZ antitumor activity, we assessed tumor tissue biomarkers by immunohistochemistry (Figure 3). Tumors from RuMTZ-treated mice displayed increased immunoreactivity for cleaved caspase-3 (Figure 3A), γH2AX (Figure 3B), and ERK1/2 (MAPK/ERK pathway component; Figure 3C) compared with the vehicle group. These changes are consistent with engagement of apoptotic processes, activation of DNA damage response signaling, and modulation of MAPK/ERK-related signaling in association with RuMTZ treatment.

**Figure 3:**
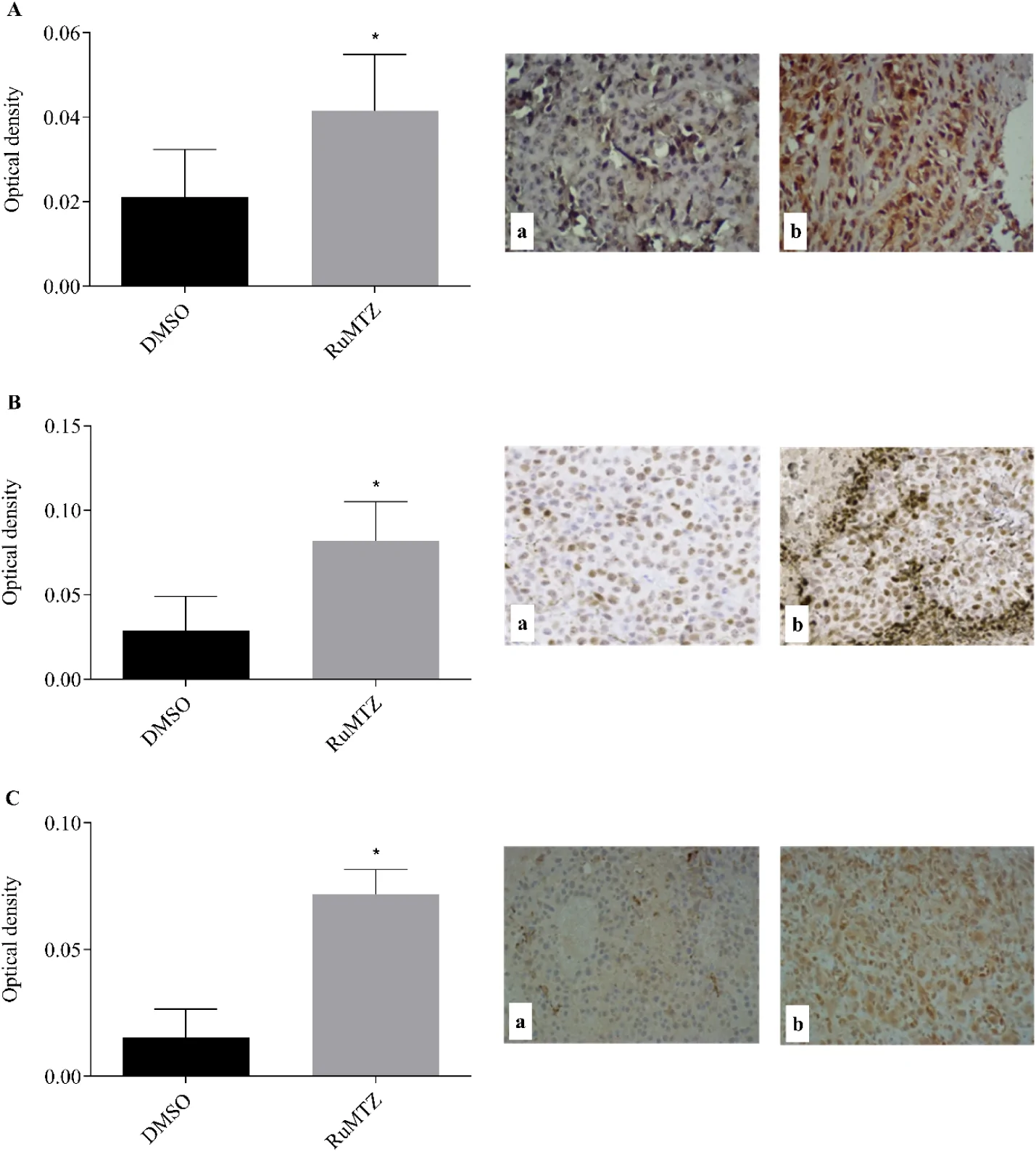
Optical density values of cleaved caspase-3 (A), γH2AX (B), and p44/42 MAPK (ERK 1/2) (C) immunostaining in tumor tissues of C57BL/6 mice after 5 days of treatment. Immunostaining with DAB, 3,3-diaminobenzidine (brown color) counterstaining with hematoxylin. DMSO – dimethyl sulfoxide (1%); RuMTZ – [Ru­(mtz)­(dppe)_2_]­PF_6_-2-mercaptothiazoline-di-1,2-bis­(diphenylphosphine)­ethanoruthenium­(II) hexafluorophosphate (5 mg/kg). Values are expressed as mean ± standard deviation (n = 3). a – DMSO; b – RuMTZ. *Significantly different from the DMSO group (*p*< 0.05).

Caspase-3 has been identified as a key mediator of apoptosis, being activated by both the extrinsic and intrinsic pathways. Given the critical role of caspase-3 in apoptotic flux, its deficiency may disrupt apoptosis, resulting in carcinogenesis. Accordingly, the overexpression of caspase-3, as an apoptosis-inducing agent, has clinical significance.^31^ This enzyme has therefore been considered a therapeutic target for combating apoptosis-related diseases, including various cancers. The design of caspase-3-activating or -inhibiting molecules has been one of the common strategies in this context.^32^ Thus, the data obtained indicate that the antimelanoma effect of RuMTZ is associated with a caspase-dependent apoptotic pathway.

The results also showed increased expression of γH2AX induced by RuMTZ. Phosphorylation of H2AX to γH2AX is a critical event in the cellular response to DNA strand breaks and serves as an indicator of genomic instability.^33,34^ H2AX phosphorylation acts as a DNA damage signal, triggering downstream pathways that govern cell cycle checkpoints, apoptosis, and the cellular fate decision between repair and cell death.^35^

H2AX plays a pivotal role in the regulation of apoptosis. This protein undergoes specific post-translational modifications in response to apoptosis-inducing stimuli, which are essential for the initiation and execution of programmed cell death.^36,37^ Accordingly, the antimelanoma effect of RuMTZ is due, at least in part, to the intricate involvement of H2AX and apoptosis, as evidenced by the increased expression of cleaved caspase-3. It should be emphasized that these results are consistent with those previously obtained by our research group in melanoma cell cultures treated with RuMTZ.^13^ Recent therapeutic strategies have highlighted H2AX as a key component of the DNA damage response and apoptosis pathways, positioning it as a significant target in cancer treatment.^38^

Silva and collaborators^10^ demonstrated that RuMTZ does not cause significant damage to the tertiary and secondary structures of DNA. This finding suggests that the DNA strand breaks inferred by the increase in γH2AX observed in the present study likely do not result from direct interaction with DNA. However, RuMTZ was shown to inhibit the activity of human topoisomerase IB (hTopIB), which may be attributed both to a direct action on hTopIB and to its interaction with DNA (hTopIB-DNA). In line with these observations, molecular docking results demonstrated that RuMTZ exhibits affinity for the active sites of both free hTopIB and hTopIB-DNA, which may lead to inhibition of enzymatic activity.^10^

Similarly, previous studies with ruthenium complexes bearing phosphine and mercapto ligands have reported that these compounds do not significantly alter the tertiary or secondary structures of DNA. Biophysical analyses, including viscosity measurements and electrophoretic mobility assays, indicate that binding modes such as irreversible covalent interactions and intercalation are unlikely. Instead, when interactions with DNA occur, they tend to be weak and reversible, possibly involving minor groove binding through hydrophobic, electrostatic, and van der Waals interactions. These findings suggest that the cytotoxic effects of such complexes are more likely associated with interactions with other biological targets rather than direct DNA damage. In this context, the inhibition of hTopIB by RuMTZ may contribute to the activation of DNA damage signaling pathways, as evidenced by the increased γH2AX levels observed in the present study.^30,39^

According to Takarada and colleagues,^40^ ruthenium­(II) phosphine complexes bearing mercapto ligands have been identified as potent inhibitors of hTopIB. Agents that interfere with the interaction between topoisomerase and DNA can divert enzymatic activity toward a cytotoxic pathway, either by preventing DNA binding or by stabilizing the protein–DNA complex, thereby inducing permanent DNA damage during the replication process^41^ and triggering apoptosis.^42,43^

Increased ERK1/2 immunoreactivity was also observed in melanomas from mice treated with RuMTZ. Novel drugs targeting the MAPK pathway have generated significant clinical responses in melanoma therapy, as this pathway plays a central role in melanoma pathogenesis.^44–46^ The ERK1/2 pathway is frequently deregulated in tumors due to activating mutations in *RAS* or *B-RAF*, which are commonly observed in malignant melanoma. Several studies have associated its oncogenic potential with increased cell survival, mainly through the promotion of antiapoptotic proteins. Paradoxically, an increasing number of bodies of evidence suggests that, under certain conditions, DNA damage has been reported to modulate ERK1/2 signaling and may contribute to apoptotic responses.^47–49^

DNA damage-induced cell commitment to cell cycle arrest, senescence, or apoptosis likely involves the integration of DNA damage response signaling with additional pathways governing general stress and survival responses, such as the MAPK pathway.^50^ The proapoptotic function of the MAPK/ERK pathway is well documented in apoptosis induced by DNA-damaging agents.^48^

Carvalho and colleagues^51^ demonstrated the activation of ERK 1/2-mediated apoptosis in human hepatocellular carcinoma cells (HepG2) treated with a ruthenium complex containing xanthoxylin. This compound induced ERK 1/2 phosphorylation and consequently triggered apoptosis in these cells. Accordingly, another study demonstrated that ruthenium complexes containing heterocyclic thioamides, such as MTZ, bind to DNA and inhibit cell proliferation, triggering caspase-mediated apoptosis through ERK 1/2 signaling in HepG2 cells.^52^

The findings of the present study, based on the integrated analysis of molecular markers, suggest that the RuMTZ exerted its antimelanoma effect through a coordinated series of events involving DNA damage and apoptosis. The increase in γH2AX levels indicates that RuMTZ induced DNA strand breaks, possibly through inhibition of topoisomerase IB. DNA damage may be, at least in part, related to the induction of caspase-mediated apoptosis. In addition, increased ERK1/2 immunoreactivity was observed in melanomas from mice treated with RuMTZ. However, the involvement of this pathway in the antimelanoma action of RuMTZ requires further investigation.

### 3.2 Toxicity Assessment

The organ weights of mice from different experimental groups are shown in Table 1. Animals treated with RuMTZ showed no significant differences compared with the negative control groups, indicating a lack of toxicity in nontumor tissues. Conversely, the tumor-bearing group that received the DMSO vehicle or was treated with the MTZ ligand showed significantly greater weight gain compared with the negative control group. This increase in body weight gain is related to the presence of the tumor. On the other hand, a significant reduction in body weight gain was observed in rodents treated with CDDP, indicating treatment-related toxicity (Figure 4).

**Table 1:** Organ Weight of Tumor-Bearing C57BL/6 Mice Subjected to Treatment with RuMTZ for Five Consecutive Days and the Respective Control Groups^a^

	**Organ weight (g)**				
Treatments	Liver	Kidneys	Spleen	Heart	Lung
NC	0.96 ± 0.20	0.26 ± 0.03	0.06 ± 0.02	0.10 ± 0.01	0.13 ± 0.01
DMSO	0.86 ± 0.07	0.28 ± 0.08	0.20 ± 0.02	0.12 ± 0,03	0.13 ± 0.01
MTZ	0.80 ± 0.07	0.19 ± 0.01	0.17 ± 0.01	0.10 ± 0.00	0.12 ± 0.01
RuCl_2_	0.69 ± 0.04	0.12 ± 0.07	0.07 ± 0.06	0.07 ± 0.01	0.10 ± 0.03
RuMTZ	0.86 ± 0.03	0.29 ± 0.04	0.16 ± 0.09	0.11 ± 0.01	0.13 ± 0.02
CDDP	0.44 ± 0.16^b^	0.27 ± 0.02	0.07 ± 0.11	0.11 ± 0.01	0.14 ± 0.02

a NC – negative control (tumor-free and untreated); DMSO – dimethyl sulfoxide (1%); MTZ – 2-mercaptothiazoline (0.59 mg/kg); RuCl_2_ – Ru­[Cl_2_]­dppe_2_)­PF_6_ – cis-dichloro-di-1,2-bis­(diphenylphosphine)­ruthenium­(II) (4.78 mg/kg); RuMTZ – [Ru­(mtz)­(dppe)_2_]­PF_6_ – 2-mercaptothiazoline-di-1,2-bis­(diphenylphosphine)­ethanoruthenium­(II) hexafluorophosphate (5.71 mg/kg); CDDP – cisplatin (5 mg/kg). Values are expressed as mean ± standard deviation (*n* = 5).

b Significantly different from the NC group (*p* < 0.05).

**Figure 4:**
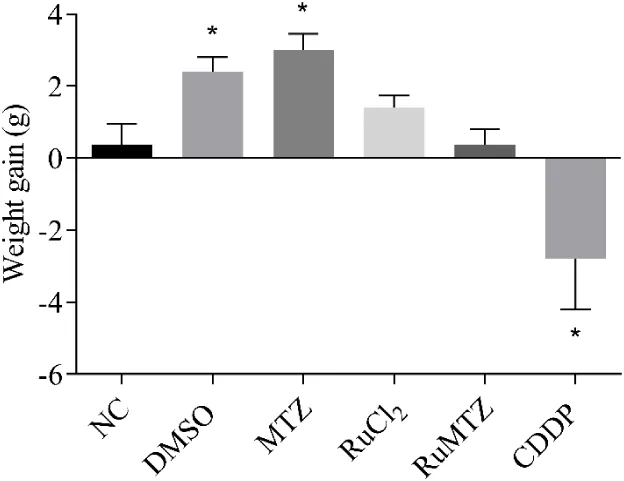
Body weight gain of tumor-bearing C57BL/6 mice subjected to treatment with RuMTZ for five consecutive days and the respective control groups. NC – negative control (tumor-free and untreated); DMSO – dimethyl sulfoxide (1%); MTZ – 2-mercaptothiazoline (0.59 mg/kg); RuMTZ – [Ru­(mtz)­(dppe)_2_]­PF_6_-2-mercaptothiazoline-di-1,2-bis­(diphenylphosphine)­ethanoruthenium­(II) hexafluorophosphate (5 mg/kg); CDDP – cisplatin (5 mg/kg). Values are expressed as mean ± standard deviation (n = 5). *Significantly different from the NC group (*p*< 0.05).

Serum levels of creatinine and ALT in rodents treated with RuMTZ were not significantly different from those observed in the negative control group, indicating the absence of nephrotoxicity and hepatotoxicity (Figure 5A and 5B, respectively). In contrast, animals in the DMSO and CDDP groups exhibited a significant increase in creatinine levels, indicating nephrotoxicity (Figure 5A). Notably, creatinine is a sensitive marker of renal function, and its elevation typically reflects kidney damage exceeding 50%. Furthermore, animals treated with CDDP showed a significant increase in serum ALT levels compared with the negative control group (Figure 5B). This finding is consistent with the significant reduction in liver weight observed in animals treated with CDDP, confirming its hepatotoxic effect.

**Figure 5:**
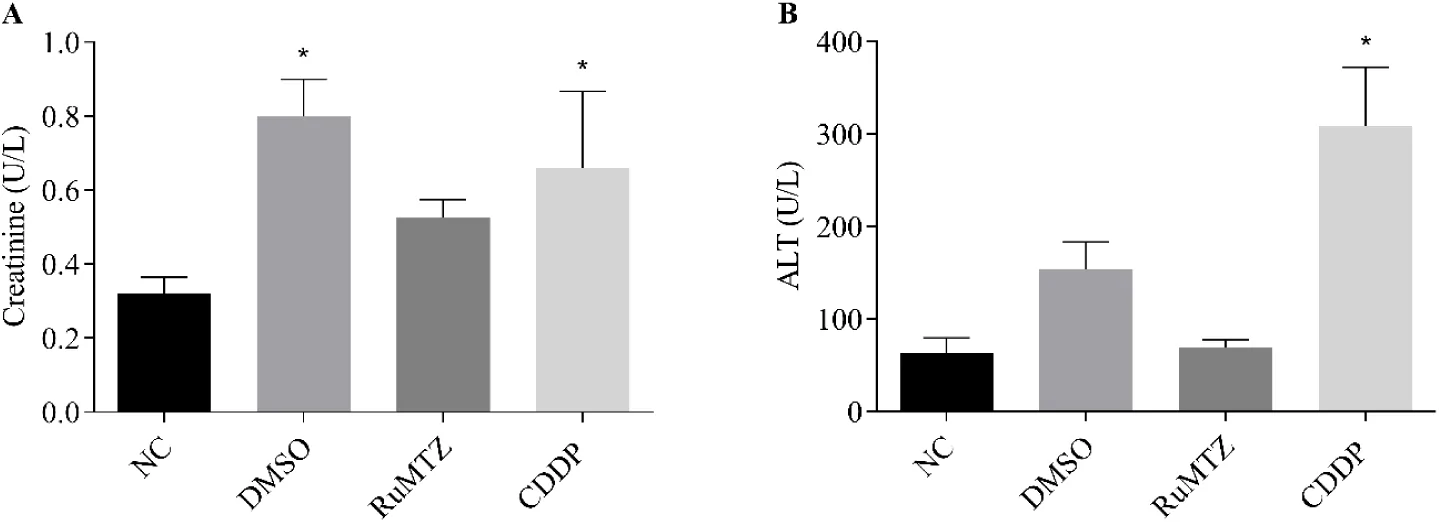
Serum levels of creatinine (A) and alanine aminotransferase (ALT) (B) in tumor-bearing C57BL/6 mice subjected to treatment with RuMTZ for five consecutive days and the respective control groups. NC – negative control (tumor-free and untreated); DMSO – dimethyl sulfoxide (1%); RuMTZ – [Ru­(mtz)­(dppe)_2_]­PF_6_-2-mercaptothiazoline-di-1,2-bis (diphenylphosphine)­ethanoruthenium­(II) hexafluorophosphate (5 mg/kg); CDDP – cisplatin (5 mg/kg). Values are expressed as mean ± standard deviation (n = 5). *Significantly different from the NC group (*p* < 0.05).

RuMTZ exhibited antimelanoma activity comparable to that of CDDP. However, unlike RuMTZ, treatment with the metallodrug showed signs of toxicity, such as body weight loss and impairment of liver and kidney functions. CDDP-induced liver damage is well-documented in the literature^53,54^ as well as nephrotoxicity, which affects approximately one-third of patients.^55^

Animals in the solvent control group also showed increased creatinine levels, indicating renal damage. Fontoura et al.^56^ reported a significant increase in serum creatinine levels in melanoma-bearing mice, indicating renal dysfunction associated with tumor progression. The abundant secretion of melanin by this type of tumor can lead to renal failure. Histopathological analyses revealed deposits of melanin and immune complexes in renal tubules and glomerular basement membranes, causing structural changes such as basement membrane thickening and inflammation, ultimately impairing kidney function.

Table 2 presents the frequencies of MNPCE observed in the bone marrow of tumor-bearing C57BL/6 mice subjected to treatment with RuMTZ for five consecutive days and the respective control groups. As a consequence of cytotoxicity observed in the bone marrow of animals treated with RuMTZ and CDDP, it was not possible to analyze micronuclei.

**Table 2:** Frequencies of Micronucleated Polychromatic Erythrocytes (MNPCE) and the Ratio of Polychromatic Erythrocytes (PCE) to Total Erythrocytes (TE) Obtained from Bone Marrow Cells from Tumor-Bearing C57BL/6 Mice Subjected to Treatment with RuMTZ for Five Consecutive Days and the Respective Control Groups^a^

Treatments	MNPCE^b^	PCE/TE^c^
NC	11.0 ± 3.46	0.75 ± 0.05
DMSO	5.2 ± 0.84	0.76 ± 0.10
RuMTZ	-	0.41 ± 0.19^d^
CDDP	-	0.52 ± 0.09^d^

a NC – Negative control (tumor-free and untreated); DMSO – dimethyl sulfoxide (1%); RuMTZ – [Ru­(mtz)­(dppe)_2_]­PF_6_ – 2-mercaptothiazoline-di-1,2-bis­(diphenylphosphine)­ethanoruthenium­(II) hexafluorophosphate (5 mg/kg); CDDP – cisplatin (5 mg/kg).

b 2000 polychromatic erythrocytes per animal were analyzed.

c 500 erythrocytes per animal were analyzed.

d Significantly different from the NC group (*p* < 0.05).

Treatments with RuMTZ, as well as with CDDP, resulted in myelosuppression, as evidenced by the absence of erythrocytes. A similar effect was observed in rodents treated with ruthenium complexes containing lapachol and lawsone ligands.^22^ Disruptions in bone marrow cell production have also been previously reported for CDDP.^57^ Due to their proliferating nature, bone marrow cells are more sensitive to clastogenic chemicals and more susceptible to DNA damage.^58^ The mechanisms underlying CDDP-induced myelosuppression are associated with *(i)* its ability to bind DNA and form intra- and interstrand DNA adducts, thereby interfering with DNA transcription and replication, and *(ii)* glutathione depletion, leading to increased oxidative stress.^59^ Although RuMTZ showed a relatively favorable short-term renal and hepatic safety profile, the observed bone marrow alterations indicate that hematological toxicity may represent an important limitation of the compound. Future studies should be conducted to better understand the action of RuMTZ on bone marrow.

## 4 Conclusions

In conclusion, this study provides the first in vivo evaluation of the RuMTZ complex in an immunocompetent B16 melanoma model. RuMTZ exhibited marked antimelanoma activity in C57BL/6 mice, achieving tumor growth inhibition comparable to cisplatin under the tested conditions while showing a more favorable systemic profile in terms of body weight and liver/kidney function markers. Tumor biomarker analyses revealed increased cleaved caspase-3 and γH2AX, together with altered ERK1/2 immunoreactivity, suggesting the involvement of apoptosis, DNA damage response signaling, and MAPK/ERK-related pathways. These findings are consistent with prior in vitro observations and with previous reports indicating that RuMTZ can inhibit hTopIB, although additional studies are required to establish in vivo target engagement and clarify the mechanistic sequence. Importantly, bone marrow toxicity was observed in RuMTZ-treated animals, preventing micronucleus scoring under the current dosing schedule and highlighting myelosuppression as a relevant limitation and safety concern to be addressed in future work. Further studies should therefore focus on defining the therapeutic window (dose and schedule optimization), evaluating recovery and longer-term safety, and expanding mechanistic validation and pharmacological characterization to support translational development.

## #

Universidade do Estado do Amapá, Macapá, Amapá 68900-070, Brasil
